# LATE ELONGATED HYPOCOTYL regulates photoperiodic flowering via the circadian clock in *Arabidopsis*

**DOI:** 10.1186/s12870-016-0810-8

**Published:** 2016-05-20

**Authors:** Mi-Jeong Park, Young-Ju Kwon, Kyung-Eun Gil, Chung-Mo Park

**Affiliations:** Department of Chemistry, Seoul National University, Seoul, 151-742 Korea; Plant Genomics and Breeding Institute, Seoul National University, Seoul, 151-742 Korea

**Keywords:** *Arabidopsis thaliana*, Circadian clock, Flowering time, LHY, Photoperiod

## Abstract

**Background:**

Plants constantly monitor changes in photoperiod or day length to trigger the flowering cycle at the most appropriate time of the year. It is well established that photoperiodic flowering is intimately associated with the circadian clock in *Arabidopsis*. In support of this notion, many clock-defective mutants exhibit altered photoperiodic sensitivity in inducing flowering. LATE ELONGATED HYPOCOTYL (LHY) and its functional paralogue CIRCADIAN CLOCK ASSOCIATED 1 (CCA1) constitute the core of the circadian clock together with TIMING OF CAB EXPRSSION 1 (TOC1). While it is known that TOC1 contributes to the timing of flowering entirely by modulating the clock function, molecular mechanisms by which LHY and CCA1 regulate flowering time have not been explored.

**Results:**

We investigated how LHY and CCA1 regulate photoperiodic flowering through molecular genetic and biochemical studies. It was found that LHY-defective mutants (*lhy-7* and *lhy-20*) exhibit accelerated flowering under both long days (LDs) and short days (SDs). Consistent with the accelerated flowering phenotypes, gene expression analysis revealed that expression of the floral integrator *FLOWERING LOCUS T* (*FT*) is up-regulated in the *lhy* mutants. In addition, the expression peaks of *GIGANTEA* (*GI*) and *FLAVIN-BINDING, KELCH REPEAT, F-BOX PROTEIN 1* (*FKF1*) genes, which constitute the clock output pathway that is linked with photoperiodic flowering, were advanced by approximately 4 h in the mutants. Furthermore, the up-regulation of *FT* disappeared when the endogenous circadian period is matched to the external light/dark cycles in the *lhy-7* mutant. Notably, whereas CCA1 binds strongly to *FT* gene promoter, LHY does not show such DNA-binding activity.

**Conclusions:**

Our data indicate that the advanced expression phases of photoperiodic flowering genes are associated with the clock defects in the *lhy* mutants and responsible for the reduced photoperiodic sensitivity of the mutant flowering, demonstrating that LHY regulates photoperiodic flowering via the circadian clock, similar to what has been shown with TOC1. It is notable that while LHY regulates photoperiodic flowering in a similar manner as with TOC1, the underlying molecular mechanism would be somewhat distinct from that exerted by CCA1 in *Arabidopsis*.

**Electronic supplementary material:**

The online version of this article (doi:10.1186/s12870-016-0810-8) contains supplementary material, which is available to authorized users.

## Background

The appropriate timing of flowering is critical for reproductive success in plants. Since the transition from the vegetative to the reproductive phases is irreversible, plants precisely coordinate endogenous developmental signals and environmental cues, such as changes in photoperiod, light quality and quantity, and temperature, to optimize the timing of flowering [1–3]. Both the endogenous and environmental signals are incorporated into flowering genetic pathways via the floral integrators, such as *FLOWERING LOCUS T* (*FT*) and *SUPPRESSOR OF CONSTANS OVEREXPRESSION 1* (*SOC1*) [4, 5].

Photoperiod is a major environmental cue that profoundly affects floral induction [2, 3, 6]. Plants monitor photoperiodic changes to anticipate seasonal changes. CONSTANS (CO), which is a B-box zinc finger transcription factor [7], plays a central role in photoperiodic flowering by activating *FT* expression [8, 9]. Accordingly, CO-defective mutants and CO-overexpressing plants exhibit photoperiod-insensitive flowering phenotypes [10, 11].

The photoperiod-sensitive *FT* induction is mediated by the distinct accumulation peak of CO in late afternoon under long days (LDs), which is shaped by the coordinated actions of several ubiquitin/proteasome-dependent pathways [12]. A small group of E3 ubiquitin ligases and photoreceptors modulate the CO stability. The light signaling mediator CONSTITUTIVE PHOTOMORPHOGENIC 1 (COP1) degrades CO at night [13, 14]. In the light phase of the day, two opposing regulations occur through the actions of HIGH EXPRESSION OF OSMOTICALLY RESPONSIVE GENES 1 (HOS1) and FLAVIN-BINDING, KELCH REPEAT, F-BOX 1 (FKF1) E3 ubiquitin ligases. HOS1 degrades CO in the morning, and FKF1 stabilizes CO in late afternoon [15, 16]. The sequential actions of HOS1 and FKF1 contribute to the maintenance of CO accumulation at a basal level in the morning but at a high level in later afternoon, and thus *Arabidopsis* flowering is induced only under LDs [6]. Meanwhile, PHYTOCHROME B mediates CO degradation, but PHYTOCHROME A and CRYTOCHROME photoreceptors mediate CO stabilization [12]. It is notable that CO accumulation occurs at the specific time phase of the day under LDs, necessitating that photoperiodic flowering would be closely linked with the circadian clock [2, 3, 17].

Many clock-defective *Arabidopsis* mutants exhibit alterations in the photoperiodic sensitivity of flowering time, supporting the intimate linkage between the clock and photoperiodic flowering [18–22]. In addition, the circadian clock regulates the rhythmic expression of photoperiodic flowering genes, such as *CO*, *FKF1*, *GIGANTEA* (*GI*), and *CYCLING DOF FACTOR 1* (*CDF1*) [23–26]. The clock allows the high-level expression of *CO* gene occurs in the light only under LDs [23]. The clock-controlled peak of *FKF1* and *GI* expression in the LD afternoon renders FKF1-GI complex to be formed, which is crucial for CO accumulation [16]. On the other hand, the prevention of *CO* and *FT* expression by CDFs occurs in the morning through the clock function [26, 27]. It has been shown that the early flowering phenotypes of short-period plants, such as TIMING OF CAB EXPRESSION 1 (TOC1)-defective mutant (*toc1*) and CASEIN KINASE II BETA SUBUNIT 4 (CKB4)-overexpressing plants, are caused by the advanced expression peaks of photoperiodic flowering genes [28, 29].

While the altered flowering phenotypes of *toc1* mutant and CKB4-overexpressing plants are entirely caused by clock defects, clock-independent control of photoperiodic flowering by clock components has also been proposed [30–32]. For instance, GI, which plays a role in regulating clock progression [33], activates *FT* expression by directly binding to its gene promoter independent of CO [30]. It has recently been reported that SENSITIVITY TO RED LIGHT REDUCED 1 (SRR1), which is required for normal oscillator function [34], regulates floral transition in a CO-independent manner [31]. Similarly, DE-ETIOLATED 1 (DET1), which is a transcriptional corepressor important for clock progression, acts as a floral repressor [32]. It is notable that GI, SRR1, and DET1 regulate flowering time independent of their roles in the clock function. In addition, they do not require CO, the central promoter of photoperiodic flowering.

LATE ELONGATED HYPOCOTYL (LHY) and its functional paralogue CIRCADIAN CLOCK ASSOCIATED 1 (CCA1) constitute the central oscillator in *Arabidopsis* [20, 35, 36]. *Arabidopsis* mutants that are defective in LHY and CCA1 exhibit early flowering even under non-inductive conditions [20, 22, 37]. It has been shown that CCA1 regulates flowering time by binding to the *SOC1* gene promoter [38]. However, it has not been explored at the molecular level how LHY regulates flowering time.

In this work, with an aim of clarifying the molecular mechanism by which LHY regulates flowering time, we performed molecular genetic and biochemical studies on LHY-defective mutants (*lhy-7* and *lhy-20*). Notably, the expression peaks of photoperiodic flowering genes were shifted earlier in the *lhy* mutants. We found that the advanced expression phases of photoperiodic flowering genes are associated with the clock defects in the mutants and underlie the reduced photoperiodic sensitivity of the mutant flowering. By matching the external light/dark cycles to the endogenous circadian period, the early flowering phenotype of the mutants was rescued. Interestingly, CCA1 binds strongly to the *FT* gene promoter, but LHY does not exhibit such DNA-binding activity. Our data indicate that while LHY regulates the timing of flowering entirely via the circadian clock like TOC1, CCA1 would be functionally distinct from TOC1 and LHY in regulating photoperiodic flowering.

## Results

### Loss-of-function *lhy* mutants exhibit early flowering under both LDs and SDs

**Fig. 1:**
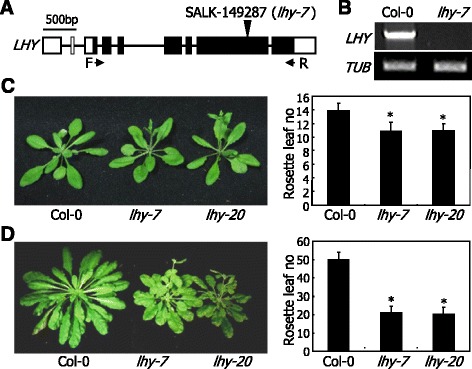
LHY-defective mutants exhibit early flowering under both LDs and SDs. **a** Isolation of an LHY-defective mutant (*lhy-7*). It was isolated from a pool of T-DNA insertional lines deposited in the *Arabidopsis* Biological Resource Center (Ohio State University, OH). bp, base pair. F and R, forward and reverse primers, respectively, used to examine the expression of *LHY* gene. **b** Lack of *LHY* gene expression in *lhy-7* mutant. Gene expression was examined by RT-PCR. A tubulin gene (*TUB*) was used as control for constitutive expression. **c** Flowering phenotypes of *lhy* mutants under LDs. The previously reported *lhy-20* mutant was also included in the assays [39]. Plants were grown until flowering in soil under LDs (16-h light and 8-h dark) (*left panel*). Rosette leaf numbers of 20 plants were averaged and statistically treated using Student *t*-test (*P < 0.01) for each plant genotype (*right panel*). *Bars* indicate standard error of the mean (SE). **d** Flowering phenotypes of *lhy* mutants under SDs. Plants were grown until flowering in soil under SDs (8-h light and 16-h dark) (*left panel*). Flowering times were measured as described in (**c**) (*right panel*)

To investigate the functional roles of LHY in photoperiodic flowering, we examined the flowering phenotypes of LHY-defective mutants. We also aimed to clarify the molecular mechanisms by which LHY regulates flowering time: whether LHY affects flowering time entirely by modulating the circadian rhythms like TOC1 [28] or regulating the expression of specific flowering genes like GI [30] or both. In additional to the previously reported *lhy-20* mutant [39], we also isolated a T-DNA insertional loss-of-function mutant, which was designated *lhy-7* (Fig. 1a). The *lhy-7* mutant contains a single copy of T-DNA insertion in the sixth exon of *LHY* gene. Gene expression analysis confirmed lack of *LHY* transcription in the *lhy-7* mutant (Fig. 1b).

It has been reported that the loss-of-function *lhy-20* mutant exhibit a shortening of the circadian period [39, 40]. We investigated the circadian period of the *lhy-7* mutant by examining the rhythmic expression patterns of two representative clock output genes, *COLD, CIRCADIAN RHYTHM, AND RNA BINDING 2* (*CCR2*) and *CHLOROPHYLL A/B-BINDING PROTEIN 2* (*CAB2*) [41, 42]. We found that the *lhy-7* mutant also exhibits advanced peak expressions of the *CCR2* and *CAB2* genes compared to those observed in Col-0 plants (Additional file 1), as has been observed with other LHY-defective mutants [20, 37, 39, 40].

We next examined the flowering phenotypes of the *lhy-7* and *lhy-20* mutants under different daylengths by counting the number of rosette leaves at bolting. Both the *lhy* mutants showed accelerated flowering under LDs (Fig. 1c). The early flowering phenotypes of the mutants were more prominent under SDs (Fig. 1d). The reduced photoperiodic sensitivity of the *lhy* flowering is similar to what has been observed with LHY-defective mutants in other ecotypes [20, 37], showing that the altered flowering phenotypes are associated with LHY function. Since the flowering phenotypes of the *lhy-7* and *lhy-20* mutants were similar each other, we chose the former for subsequent molecular assays.

### Expression patterns of flowering genes are altered in *lhy-7* mutant

**Fig. 2:**
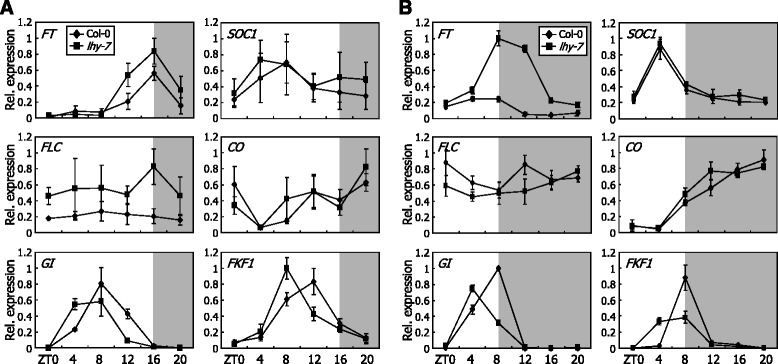
Expression patterns of flowering genes are altered in *lhy-7* mutant*.* Plants were grown under either LDs or SDs for 10 days on 1/2 X Murashige and Skoog-agar plates (hereafter, referred to as MS-agar plates) before harvesting whole plant materials for total RNA extraction. Transcript levels were examined by quantitative real-time RT-PCR (qRT-PCR). Biological triplicates were averaged and statistically treated using Student *t*-test. *Bars* indicate SE. ZT, zeitgeber time. **a** Expression of flowering time genes under LDs. **b** Expression of flowering time genes under SDs

To obtain insights into the molecular mechanism by which LHY regulates flowering time, we analyzed the expression patterns of flowering genes under LDs and SDs. In LD-grown plants, the expression of *FT* and *SOC1* genes was slightly but detectably elevated in the *lhy-7* mutant (Fig. 2a), which is in good agreement with the flowering phenotype of the mutant. Under SDs, the expression of *FT* gene, but not *SOC1* gene, was markedly elevated in the *lhy-7* mutant (Fig. 2b), indicating that the *FT* induction is the major cause of the early flowering of the mutant at least under SDs.

It is known that the circadian clock is closely associated with photoperiodic flowering [1, 17, 37]. We therefore examined the expression patterns of photoperiodic flowering genes, such as *CO*, *GI*, and *FKF1*, in the *lhy-7* mutant. The amplitude and waveform of *CO* transcription were not discernibly altered in the *lhy-7* mutant under both LDs and SDs (Fig. 2a and b). In contrast, the waveforms of *GI* and *FKF1* transcription appeared with advanced shifts of the peaks in the *lhy-7* mutant under both photoperiod regimes. Considering that GI interacts with FKF1 to stabilize CO [16], it seems that the advanced expression phases of *GI* and *FKF1* under SDs would lead to an elevation of the GI-FKF1 complex formation and stabilize CO, underscoring the *FT* induction and early flowering in the *lhy-7* mutant.

We also investigated the expression patterns of flowering genes that belong to other flowering pathways, such as autonomous, thermosensory, and gibberellic acid (GA) pathways, under LDs and SDs. It was found that the expression patterns of autonomous pathway genes, such as *FLOWERING LOCUS KH DOMAIN* (*FLK*), *FVE*, and *FCAγ*, and GA pathway genes, such as *SPYNDLY* (*SPY*) and *REPRESSOR OF GA1* (*RGA1*), were not altered in the *lhy*-7 mutant under both light regimes (Additional file 2). In addition, the transcription of two floral repressor genes, which play a role in temperature-responsive flowering, was not significantly affected in the mutant. While the transcript level of *SHORT VEGETATIVE PHASE* (*SVP*) gene in the mutant was comparable to that in Col-0 plants, the transcription of *FLOWERING LOCUS M β* (*FLMβ*) gene was marginally induced in the mutant (Additional file 2). Considering the floral repressive activity of FLMβ [43], it is apparent that the early flowering phenotype of the *lhy-7* mutant is not associated with the slight induction of *FLMβ* gene.

### *FLC* gene is not related with *lhy-7* flowering

It has been reported that induction of the floral repressor *FLOWERING LOCUS C* (*FLC*) is linked with the late flowering phenotype of *cca1 lhy* double mutant grown under continuous light conditions [44]. The *FLC* gene is also up-regulated in the parental *lhy* mutant in Landsberg *erecta* (L*er*) background. We found that *FLC* expression is slightly increased in the *lhy-7* mutant under LDs (Fig. 2a). In contrast, the *FLC* expression was not discernibly elevated in the mutant under SDs (Fig. 2b), suggesting that *FLC* gene is not associated with the mutant flowering phenotype.

**Fig. 3:**
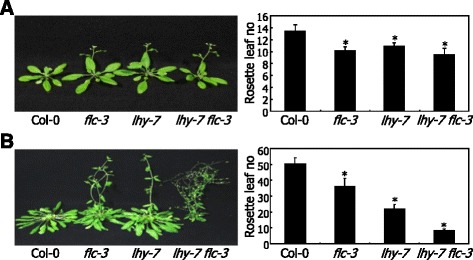
*FLC* gene is not associated with the flowering phenotype of *lhy-7* mutant. The *lhy-7* mutant was crossed with the *flc-3* mutant, resulting in *lhy-7 flc-3* double mutant. Plants were grown until flowering in soil under either LDs or SDs (*left panel*). Rosette leaf numbers of 20 plants were averaged and statistically treated using Student *t*-test (*P < 0.01) (*right panel*). *Bars* indicate SE. **a** Flowering phenotype of *lhy-7 flc-3* double mutant under LDs. **b** Flowering phenotype of *lhy-7 flc-3* double mutant under SDs

To further examine whether *FLC* gene is associated with the flowering phenotype of the *lhy-7* mutant, we crossed the *lhy-7* mutant with the FLC-defective *flc-3* mutant that exhibits early flowering, more prominently under SDs [45]. We compared the flowering time of the resultant *lhy-7 flc-3* double mutant with those of the parental mutants. The flowering phenotype of the *lhy-7 flc-3* double mutant was not discernibly different from those of the single mutants under LDs (Fig. 3a). In contrast, the *lhy-7 flc-3* double mutant flowered earlier than the single mutants under SDs (Fig. 3b), indicating that *FLC* gene is not directly associated with the flowering phenotype of the *lhy-7* mutant.

### LHY does not bind to *FT* promoter

LHY and CCA1 regulate a variety of genes by directly binding to the gene promoters [35, 38, 46]. For example, the CCA1 transcription factor represses *SOC1* expression by binding directly to the gene promoter [38]. We found that *FT* gene is significantly up-regulated in the *lhy-7* mutant (Fig. 2). It was therefore suspected that LHY might repress *FT* expression perhaps by binding to the gene promoter.

**Fig. 4:**
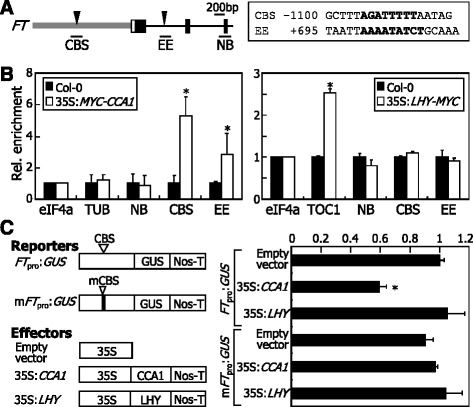
LHY does not bind to *FT* promoter. **a** Genomic structure of *FT* gene. (*Left panel*) *Gray box* indicates the gene promoter region. *Black boxes* indicate exons, and *white boxes* indicate untranslated regions. CBS, CCA1-binding sequence. EE, evening element. NB, non-binding sequence. (*Right panel*) CBS and EE sequences are listed. **b** ChIP assays on binding of CCA1 and LHY to *FT* promoter. A MYC-coding sequence was fused in-frame to the 5′ end of the CCA1-coding sequence and the 3′ end of the LHY-coding sequence, and the gene fusions were overexpressed driven by the Cauliflower Mosaic Virus (CaMV) 35S promoter in Col-0 plants, resulting in 35S:*MYC-CCA1* and 35S:*LHY-MYC*, respectively. Chromatins were prepared from 7-day-old whole plants grown on MS-agar plates and immunoprecipitated using an anti-MYC antibody. Fragmented genomic DNA was eluted from the protein-DNA complexes and subjected to quantitative PCR. Biological triplicates were averaged and statistically treated using Student *t*-test (*P < 0.01). *Bars* indicate SE. The promoter sequences of *eIF4a* and *TUB* genes were included as negative controls in the assays. The promoter sequence of *TIMING OF CAB EXPRESSION 1* (*TOC1*) gene containing EE was included as positive control [35]. **c** Suppression of *FT* transcription by CCA1. The reporter and effector constructs are illustrated (*left panel*). Transient GUS expression assays were performed using *Arabidopsis* protoplasts (*right panel*). Five measurements were averaged and statistically treated (*t*-test, *P < 0.01). *Bars* indicate SE

Nucleotide sequence analysis identified a putative CCA1-binding sequence (CBS) in the *FT* gene promoter and a potential evening element (EE) in the first intron (Fig. 4a). To examine whether LHY binds to the CBS and EE sequences, we employed chromatin immunoprecipitation (ChIP) assays using transgenic plants overexpressing a *LHY*-*MYC* gene fusion, in which a MYC-coding sequence was fused in-frame to the 3′ end of the LHY-coding sequence (Additional file 3). We also included the transgenic plants overexpressing a *MYC-CCA1* gene fusion in the assays. Both the 35S:*LHY-MYC* and 35:*MYC-CCA1* transgenic exhibited elongated hypocotyls, disruption of circadian rhythms, and suppression of *FT* expression (Additional file 4), as reported previously [19, 47], confirming that the transgenic plants are relevant for ChIP assays. Quantitative ChIP-PCR runs revealed that CCA1 binds to the CBS and EE sequence elements (Fig. 4b). In contrast, LHY did not bind to the sequence elements, while it efficiently bound to the *TOC1* gene promoter containing EE [35].

To verify the binding of CCA1 to *FT* chromatin, we performed electrophoretic mobility shift assay (EMSA) using a recombinant maltose binding protein (MBP)-CCA1 fusion protein. Consistent with the ChIP data, it was found that CCA1 binds specifically to CBS and EE sequence elements (Additional files 5 and 6).

To examine whether CCA1 binding to *FT* chromatin influences *FT* expression, we performed transient β-glucuronidase (GUS) expression assays by coexpressing the *FT* promoter-driven GUS reporter plasmid (*FT*_pro_:*GUS*) and the effector plasmids (35S:*CCA1* or 35S:*LHY*) in *Arabidopsis* protoplasts. The assays showed that CCA1 negatively regulates *GUS* expression, but LHY does not affect *GUS* expression (Fig. 4c), consistent with the ChIP data. The repressive effects of CCA1 on *GUS* expression disappeared when a reporter plasmid harboring mutations in CBS was coexpressed, indicating that the binding of CCA1 to CBS is essential for the CCA1-mediated repression of *FT* expression. These observations indicate that although LHY and CCA1 are known to be functionally redundant [20, 48], LHY is distinct from CCA1 in regulating *FT* expression.

It has been reported that *GI* is associated with the flowering phenotype of *cca1 lhy* double mutant [22]. While CCA1 is directly associated with *GI* promoter [38, 49], it is unknown whether LHY binds to *GI* promoter. ChIP assays using transgenic plants overexpressing a *LHY-MYC* gene fusion showed that LHY also binds to *GI* promoter (Additional file 7), like CCA1. Binding of both LHY and CCA1 to *GI* promoter is in harmony with the repression of *GI* expression in both *CCA1*- and *LHY*-inducible lines [50].

### Clock defects underlie the reduced photoperiodic sensitivity of *lhy* flowering

LHY is a key component of the central oscillator of plant circadian clock. LHY-defective mutants exhibit a shortened circadian rhythm of approximately 20 h compared to that of Col-0 plants ([39], this work). We found that *lhy-7* mutant exhibits early flowering with a reduced sensitivity to photoperiod. Therefore, a critical question was whether the reduced photoperiodic sensitivity of the *lhy*-*7* flowering is interconnected with the clock defects in the mutant.

**Fig. 5:**
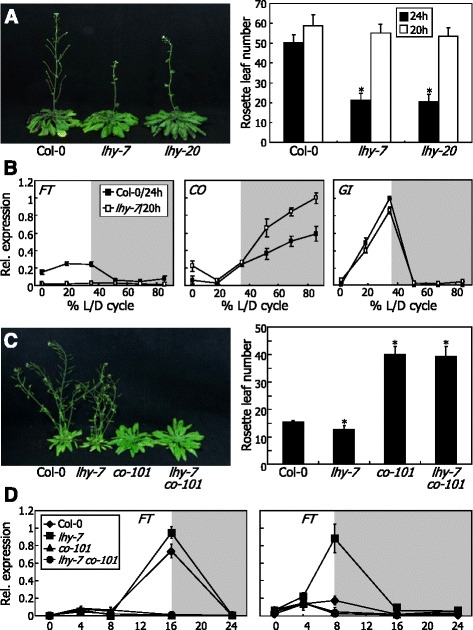
Clock defects underlie the reduced photoperiodic sensitivity of *lhy* flowering. **a** Flowering times of *lhy* mutants under SDs of differential total duration. Plants were grown until flowering under SDs of 24-h or 20-h total duration. Those grown under SDs of 20-h total duration were photographed (*left panel*). Rosette leaf numbers of 20 plants were averaged and statistically treated (*t*-test, *P < 0.01) (*right panel*). *Bars* indicate SE. **b** Expression profiles of *FT* and clock output genes in *lhy-7* mutant grown under SDs of 24-h or 20-h total duration. Transcript levels were examined by qRT-PCR. L/D, light/dark. **c** Flowering phenotype of *lhy-7 co-101* double mutant under LDs. The *lhy-7* mutant was crossed with the *co-101* mutant, resulting in *lhy-7 co-101* double mutant. Plants were grown until flowering in soil under LDs (*left panel*). Flowering times were measured as described in (**a**) (*right panel*). **d** Expression of *FT* gene in *lhy-7 co-101* double mutant grown under either LDs or SDs. Transcript levels were examined by qRT-PCR. Biological triplicates were averaged. *Bars* indicate SE

To address the question, we measured the flowering times of *lhy-7* and *lhy-20* mutants under light/dark (L/D) cycles that were matched to the endogenous circadian periods of the mutants. If the altered flowering times of the *lhy* mutants are entirely due to the clock defects, the flowering times would be restored by matching the external L/D cycles to the endogenous circadian period, as has been observed with TOC1-defective mutants [21, 28]. As expected, we found that the early flowering of the *lhy* mutants was completely annulled under SDs of 20 h (6.7 L: 13.3D) (Fig. 5a), which matches to the endogenous period of the mutants ([39], this work), indicating that LHY regulates flowering time entirely via the circadian clock.

We also analyzed the expression profiles of flowering time genes in the *lhy-7* mutant under SDs of adjusted L/D cycles. The elevation of *FT* transcript levels was not observed when the *lhy-7* mutant was grown under SDs of 20 h (Fig. 5b and Additional file 8), as has been observed with short-period plants, such as *toc1* null mutants [28, 29]. It is therefore evident that the reduced photoperiod sensitivity of the *lhy-7* flowering is caused by the clock defects.

It was found that *CO* transcription was elevated at night under the assay conditions (Fig. 5b). Since CO protein is degraded under dark conditions [13, 14], it is unlikely that the *CO* induction at night is physiologically important for the flowering phenotype of the *lhy-7* mutant. The waveform of *GI* transcription under the adjusted L/D cycles was comparable to that in Col-0 plants under SDs of 24 h. In addition, gene expression assays revealed that the expression of flowering genes, such as *FLK*, *FVE*, *FCAγ*, *SVP*, *SPY,* and *RGA1*, was not altered in the *lhy-7* mutant under both SDs of 20 and 24 h. The slight induction of *FLMβ* gene in the *lhy-7* mutant under SDs of 24 h was also observed under SDs of 20 h (Additional files 2 and 9). On the basis of the flowering times and expression patterns of flowering genes in the *lhy* mutants under adjusted L/D cycles, we concluded that LHY regulates photoperiodic flowering via the clock function.

To verify that LHY regulates flowering time through the clock-controlled CO-FT pathway, we generated *lhy-7 co-101* double mutant by crossing the *lhy-7* mutant with the CO-defective *co-101* mutant. Acceleration of flowering by the *lhy* mutation completely disappeared in the *lhy-7 co-101* double mutant under LDs (Fig. 5c). Accordingly, *FT* expression was slightly induced in the *lhy-7* mutant, but the induction was compromised in the *lhy-7 co-101* double mutant (Fig. 5d). We also examined the level of *FT* transcripts in the single and double mutants under SDs, since the early flowering phenotype of the single mutant was more prominent under this light regime (Fig. 1d). We found that the elevation of *FT* expression in the *lhy-7* mutant was completely annulled in the *lhy-7 co-101* double mutant (Fig. 5d). Together, these observations indicate that LHY-mediated regulation of photoperiodic flowering depends on CO function.

## Discussion

### LHY-mediated clock control of photoperiodic flowering

Plants sense photoperiodic changes by integrating light signals perceived by the photoreceptors and timing information provided by the circadian clock. In *Arabidopsis*, photoperiod-sensitive induction of the floral integrator *FT* is a crucial molecular event in photoperiodic flowering [6]. It is known that the coordinated action of light signals and timing information allows the CO transcription factor to accumulate specifically in late afternoon under LDs, which is a prerequisite for the LD-specific induction of *FT* gene [1–3, 12]. The circadian clock regulates CO activity at both the transcriptional and posttranscriptional levels [12, 16, 23]. At the transcriptional level, the clock shapes the rhythmic expression patterns of *CO* in a way that a high level of *CO* transcripts accumulates during the light phase under LDs [23]. At the posttranscriptional level, the circadian clock contributes to the stabilization of CO in late afternoon under LDs by modulating the expression of *GI* and *FKF1* genes [16, 17].

In this study, we demonstrated that LHY, which is a core component of the central oscillator in *Arabidopsis*, regulates photoperiodic flowering by adjusting the rhythmic expression patterns of photoperiodic flowering genes, such as *GI* and *FKF1*. We found that the expression peaks of *GI* and *FKF1* genes are shifted earlier in the *lhy-7* mutant, which is consistent with the shortened circadian period of the mutant. A plausible explanation is that the advanced phases of *GI* and *FKF1* expression in the *lhy-7* mutant would lead to an increase in the formation of GI-FKF1 complexes during the light phase, resulting in a higher-level accumulation of CO in the mutant. In support of this view, the early flowering phenotype of the mutant was completely annulled by matching the external L/D cycles to the endogenous circadian period. Under these assay conditions, the phase shift of *GI* transcription was restored and the up-regulated expression of *FT* was suppressed to a basal level in the *lhy-7* mutant. Together with the previous reports on short-period plants [28, 29], it seems that the circadian clock components, including LHY, regulates photoperiodic flowering by adjusting the expression timing of photoperiodic flowering genes.

### Common and distinct roles of LHY and CCA1 in photoperiodic flowering

LHY and CCA1 are MYB motif-containing transcription factors that function at least in part redundantly in the circadian clock [19, 20, 48]. Whereas the gain-of-function mutations of both *LHY* and *CCA1* genes disrupt circadian rhythms [19, 20], both the LHY*-* and CCA1-defective mutants exhibit shortened circadian periods [20, 37, 51]. The *cca1 lhy* double mutants show shorter circadian periods than the *cca1* or *lhy* single mutants [20, 37, 48].

On the other hand, there have been some reports supporting distinct roles of CCA1 and LHY. For instance, *LHY* overexpression does not enhance pathogen resistance, whereas *CCA1* overexpression induces pathogen resistance [52, 53]. Another example is the differential regulation of *CCA1* and *LHY* expression by CCA1 HIKING EXPEDITION (CHE) and BROTHER OF LUX ARRHYTHMON (BOA), the components of the *Arabidopsis* circadian clock. While CHE and BOA bind directly to *CCA1* gene promoter, they are not associated with *LHY* gene promoter [46, 54].

We found that CCA1, but not LHY, binds to *FT* gene promoter to repress its expression. It has been reported that CCA1 represses *SOC1* expression by binding to the gene promoter [38]. We observed that *SOC1* expression is not discernibly affected by *lhy* mutations, suggesting that LHY is not related with *SOC1* expression. It is known that LHY and CCA1 form both homodimers and heterodimers in vivo [47, 55]. One possibility is that whereas common roles of the two transcription factors would be related with the LHY-CCA1 heterodimers, their distinct roles would be exerted through the homodimers.

### Clock-independent control of photoperiodic flowering

It is now apparent that most clock components, including LHY, TOC1, and CKB4, regulate photoperiodic flowering via the clock function [28, 29]. Notably, the shorter the circadian periods of clock mutants, the earlier the flowering times of the mutants in most cases [37], supporting that the clock regulates photoperiodic flowering by modulating the rhythmic expression of photoperiodic flowering genes [1, 6, 17].

However, there are recent reports supporting that some clock components affect flowering time through clock-independent pathways. CCA1 regulates the expression of floral integrators by directly binding to the gene promoters ([38], this work). GI also binds directly to *FT* gene promoter [30]. In addition, the early flowering phenotype of DET1-defective mutants is not restored by matching the external L/D cycles to the endogenous circadian period of the mutants, indicating that DET1 negatively regulates flowering independent of its role in the clock function [32]. Together, it is likely that clock components regulate photoperiodic flowering through both CO-mediated, clock-dependent and CO-free, clock-independent pathways. It will be interesting to investigate how individual clock components regulate flowering time and how the clock-dependent and clock-independent pathways are functionally inter-connected with each other in photoperiodic flowering.

## Conclusions

We investigated how LHY regulates photoperiodic flowering by performing molecular genetic and biochemical studies. LHY regulates photoperiodic flowering entirely via the circadian clock. In the LHY-defective mutants, the early flowering phenotypes and the shifted phases of photoperiodic flowering gene expression were recovered by matching the external L/D cycles to the endogenous circadian periods of the mutants. It is notable that the mechanism by which LHY regulates photoperiodic flowering is somewhat distinct from that exerted by CCA1. Our findings would contribute to better understanding of how the clock function is associated with flowering time control in response to photoperiodic signals.

## Methods

### Plant genotypes and growth conditions

*Arabidopsis thaliana* lines used were in the Columbia (Col-0) background, unless specified otherwise. *Arabidopsis* plants were grown either in soil or on 1/2 X Murashige and Skoog (MS)-agar plates (hereafter referred to as MS-agar plates) at 23 °C under either LDs (16-h light and 8-h dark) or SDs of 24 h (8 L: 16D) or 20 h (6.7 L: 13.3D) total duration. White light illumination (120 μmol photons m^−2^s^−1^) was provided by fluorescent FLR40D/A tubes (Osram, Seoul, Korea).

T-DNA insertional gene knockout mutants *lhy-20*, *flc-3*, and *co-101* have been described previously [39, 45, 56]. The *LHY*-deficient *lhy-7* mutant (SALK-149287) was isolated from a T-DNA insertional mutant pool deposited in the *Arabidopsis* Information Resource (TAIR, Ohio State University, OH). Homozygotic lines were obtained by selection for three or more generations and analysis of segregation ratios. Lack of gene expression in the mutants was verified by RT-PCR before use.

A MYC-coding sequence was fused in-frame to the 5′ end of the CCA1-coding sequence or to the 3′ end of the LHY-coding sequence, and the gene fusions were subcloned into the pBA002 vector under the control of the Cauliflower Mosaic Virus (CaMV) 35S promoter. The expression constructs were transformed into Col-0 plants, resulting in 35S:*MYC-CCA1* and 35S:*LHY-MYC*, respectively. Overexpression of the transgenes was verified by quantitative real-time RT-PCR (qRT-PCR).

### Gene expression assay

Extraction of total RNA samples from appropriate plant materials and qRT-PCR conditions have been described previously [57]. Total RNA samples were pretreated with RNase-free DNase to eliminate contaminating genomic DNA before use.

RNA sample preparation, reverse transcription, and quantitative PCR were conducted according to the criteria that have been proposed to ensure reproducible and accurate measurements [58].

qRT-PCR reactions were performed in 96-well blocks with an Applied Biosystems 7500 Real-Time PCR System (Foster City, CA) using the SYBR Green I master mix in a reaction volume of 20 μl. The PCR primers were designed using the Primer Express Software installed in the system and listed in Additional file 10. The two-step thermal cycling profile employed was 15 s at 94 °C and 1 min at 68 °C. An *eIF4A* gene (At3g13920) was included in the reactions as internal control to normalize the variations in the amounts of cDNA used. All the qRT-PCR reactions were performed in biological triplicates using RNA samples extracted from three independent plant materials grown under identical conditions. The comparative ΔΔC_T_ method was employed to evaluate relative quantities of each amplified product in the samples. The threshold cycle (C_T_) was automatically determined for each reaction using the default parameters of the system. The specificity of PCR reactions was determined by melt curve analysis of the amplified products using the standard method installed in the system.

### ChIP assay

ChIP assays were performed, essentially as described previously [59], in biological triplicates using three independent plant materials grown under identical conditions. Seven-day-old 35S:*MYC-CCA1* and 35S:*LHY-MYC* transgenic plants grown on MS-agar plates were vacuum-infiltrated with 1 % (v/v) formaldehyde for cross-linking and ground in liquid nitrogen after quenching the cross-linking process. Chromatin preparations were sonicated into 0.5- to 1-kb fragments. An anti-MYC antibody (Millipore, Billerica, MA) was added to the chromatin solution, which was precleared with salmon sperm DNA/protein G agarose beads (Roche, Indianapolis, IN). The precipitates were eluted from the beads. Cross-links were reversed, and residual proteins were removed by incubation with proteinase K. DNA was recovered using the QIAquick PCR purification kit (Qiagen, Valencia, CA). Quantitative PCR was performed to determine the amounts of genomic DNA enriched in the chromatin preparations. The primers used are listed in Additional file 10.

### Transient expression assays in *Arabidopsis* protoplasts

In the reporter vector, a 2-kb promoter sequence of *FT* gene was transcriptionally fused to the *β-glucuronidase* (*GUS*) gene. The GUS reporter construct harboring a mutated CCA1-binding sequence (CBS) within the *FT* promoter was used to investigate the effects of CBS on the binding of CCA1 and LHY to the *FT* promoter. The CCA1- and LHY-coding sequences were subcloned under the control of the CaMV 35S promoter in the effector vector. The reporter and effector vectors were cotransfected into *Arabidopsis* mesophyll protoplasts by the polyethylene glycol (PEG)-calcium transfection method [60]. The CaMV 35S promoter-luciferase construct was also cotransfected as internal control. GUS activity was measured by a fluorometric method as described previously [61]. Luciferase activity assay was performed using the Luciferase Assay System kit (Promega, Madison, WI). GUS activities were normalized by luciferase activities.

### Flowering time measurement

Plants were grown in soil at 23 °C under either long days of 24 h or short days of 24 h (8 L: 16D) or 20 h (6.6 L: 13.4D) total duration until flowering. Numbers of rosette leaves at bolting were counted, and 20 countings were averaged for each measurement.

### Circadian rhythm measurement

Plants were entrained to long day cycles and then transferred to continuous light conditions for 3 days. To trace the circadian rhythm, whole plant materials were harvested at appropriate zeitgeber time (ZT) points for total RNA extraction. Gene transcript levels were measured by qRT-PCR.

### Preparation of recombinant MBP-CCA1 fusion protein

A CCA1-coding sequence was subcloned into the pMAL-c2X *Escherichia coli* (*E. coli*) expression vector (NEB, Ipswich, MA) harboring a maltose binding protein (MBP)-coding sequence. Recombinant MBP-CCA1 fusion protein was produced in *E. coli* Rosetta2 (DE3) pLysS strain (Novagen, Madison, WI). Harvested cells were resuspended in MBP buffer (20 mM Tris-HCl, pH 7.4, 200 mM NaCl, 1 mM EDTA, 10 mM 2-mercaptoethanol, 1 mM PMSF, and protease inhibitor cocktail (Roche, Indianapolis, IN)). Cell lysates were prepared by running three cycles of freezing and thawing followed by centrifugation. The fusion proteins were affinity-purified as described previously [62].

### EMSA

EMSA was performed using recombinant MBP-CCA1 fusion protein, as described previously [63]. DNA fragments were end-labeled with γ-^32^P[dATP] using T4 polynucleotide kinase (Takara, Kyoto, Japan). Labeled probes were incubated with 100 ng of MBP or MBP-CCA1 fusion protein for 30 min at room temperature in binding buffer (10 mM Tris-HCl, pH 7.6, 50 mM NaCl, 1 mM EDTA, 5 mM DTT, 5 % glycerol) supplemented with 100 ng poly(dI-dC) in the presence or absence of competitor DNA fragments. The reaction mixtures were resolved on 6 % non-denaturing polyacrylamide gel at 100 V for 1 h. The gels were dried on Whatman 3 MM paper and exposed to X-ray film.

## Additional files

Additional file 1: Circadian rhythms in *LHY-*defective mutants. Ten-day-old plants grown on ½ X Murashige and Skoog-agar plates (hereafter, referred to as MS-agar plates) under long days (LDs, 16-h light and 8-h dark) were transferred to continuous light conditions at dawn (upper diagram). DAC, days after cold imbibition. Whole plants materials were harvested at the indicated zeitgeber time (ZT) points for total RNA extraction (lower panel). Rhythmic expression of *CHLOROPHYLL A/B-BINDING PROTEIN 2* (*CAB2*) and *CAROTENOID AND CHLOROPLAST REGULATION 2* (*CCR2*) genes, which exhibit circadian rhythmic expression patterns [41, 42], was examined by quantitative real-time RT-PCR (qRT-PCR). Biological triplicates were averaged. Bars indicate standard error of the mean. Two *LHY*-defective mutants (*lhy-7* and *lhy-20*) were examined. (PDF 157 kb) Additional file 2: Expression of flowering time genes in *lhy-7* mutant. Plants were grown under either LDs or short days (SDs, 8-h light and 16-h dark) for 10 days on MS-agar plates. Whole plants were harvested at ZT 8 for total RNA extraction. Transcript levels were examined by qRT-PCR. Biological triplicates were averaged and statistically treated using Student *t*-test (**P* < 0.01). Bars indicate standard error of the mean. (PDF 131 kb) Additional file 3: Levels of *LHY* and *CCA1* transcripts in 35S:*LHY-MYC* and 35S:*MYC-CCA1* transgenic plants, respectively. Ten-day-old whole plants grown on MS-agar plates under LDs were harvested for total RNA extraction at the indicated ZT points. Transcript levels were examined by qRT-PCR. Biological triplicates were averaged and statistically treated (*t*-test, **P* < 0.01). Bars indicate standard error of the mean. (PDF 121 kb) Additional file 4: Functionality of 35S:*MYC-CCA1* and 35S:*LHY-MYC* transgenic plants. A and B. Elongated hypocotyls. Plants were grown on MS-agar plates for 5 days under either LDs (A) or SDs (B). Measurements of 20 seedlings were averaged and statistically treated (*t*-test, **P* < 0.01). Bars indicate standard error of the mean. C. Disruption of circadian rhythms. Expression patterns of *CCR2* gene were examined as described in Additional file 1. Bars indicate standard error of the mean. D. Suppression of *FT* transcription. Plants were grown under LDs for 10 days on MS-agar plates. Whole plants were harvested at ZT16 for total RNA extraction. Transcript levels were examined as described in Additional file 2. Bars indicate standard error of the mean. (PDF 195 kb) Additional file 5: Recombinant proteins used for electrophoretic mobility shift assay (EMSA). Recombinant maltose-binding protein (MBP) and MBP-CCA1 fusion protein were prepared in *E. coli* cells and affinity-purified. Protein quality was verified by running on 10 % SDS-PAGE and Coomassie brilliant blue staining. The arrow and arrowhead indicate full-size MBP and MBP-CCA1 proteins, respectively. SM, size marker. kDa, kilodalton. (PDF 263 kb) Additional file 6: EMSA on binding of MBP and MBP-CCA1 proteins to conserved sequences in *FT* locus. Recombinant MBP and MBP-CCA1 fusion proteins were prepared as described in Additional file 5. Radio-labelled CCA1-binding sequence (CBS) and evening element (EE) DNA fragments, which were described in Fig. 4a, were used. A. EMSA on MBP binding to DNA fragments. (+) and (−) indicate assays with or without MBP protein. Note that MBP alone does not bind to the CBS and EE sequences. B. EMSA on MBP-CCA1 binding to DNA fragments. The core sequences of CBS and EE were mutated, resulting in mCBS and mEE, respectively. Excess amounts (50X, 100X) of unlabeled DNA fragments were added as competitors. (PDF 182 kb) Additional file 7: ChIP assays on LHY binding to *GI* promoter. Chromatins were prepared from 7-day-old whole plants grown on MS-agar plates and immunoprecipitated using an anti-MYC antibody. Fragmented genomic DNA was eluted from the protein-DNA complexes and subjected to quantitative PCR. Biological triplicates were averaged and statistically treated using Student *t*-test (**P* < 0.01). Bars indicate standard error of the mean. GI (NB) amplifies a downstream sequence region of *GI* gene, and GI (CBS) amplifies a sequence region containing CBS in the *GI* promoter, which has been described previously [38]. (PDF 122 kb) Additional file 8: Expression of flowering genes in *lhy-7* mutant under short days of 20-h total duration. Plants were grown for 10 days under short-day cycles of either 24-h (8-h light and 16-h dark) or 20-h (6.7-h light and 13.3-h dark) total duration. Whole plant materials were harvested throughout the % light-dark (L/D) cycles. Transcript levels were examined by qRT-PCR in Col-0 plants (A) and *lhy-7* mutant (B). Biological triplicates were averaged. Bars indicate standard error of the mean. h, hour. (PDF 133 kb) Additional file 9: Expression of flowering genes in *lhy-7* mutant under SDs of 20-h total duration. Plants were grown for 10 days under short-day cycles of 20-h total duration (6.7-h light and 13.3-h dark). Whole plant materials were harvested for total RNA extraction. Transcript levels were examined by qRT-PCR. Biological triplicates were averaged and statistically treated using Student *t*-test (**P* < 0.01). Bars indicate standard error of the mean. (PDF 130 kb) Additional file 10: Primers used in qRT-PCR, RT-PCR, and ChIP-qPCR. F, forward primer; R, reverse primer. (PDF 90 kb)

## Abbreviations

BOA: BROTHER OF LUX ARRHYTHMO
CAB2: CHLOROPHYLL A/B-BINDING PROTEIN 2
CBS: CCA1-binding sequence
CCA1: CIRCADIAN CLOCK ASSOCIATED 1
CCR2: COLD, CIRCADIAN RHYTHM, AND RNA BINDING 2
CDF1: CYCLING DOF FACTOR 1
CHE: CCA1 HIKING EXPEDITION
ChIP: chromatin immunoprecipitation
CKB4: CASEIN KINASE II BETA SUBUNIT 4
CO: CONSTANS
COP1: CONSTITUTIVE PHOTOMORPHOGENIC 1
DET1: DE-ETIOLATED 1
EE: evening element
EMSA: electrophoretic mobility shift assay
FKF1: FLAVIN-BINDING, KELCH REPEAT, F-BOX PROTEIN 1
FLC: FLOWERING LOCUS C
FLK: FLOWERING LOCUS KH DOMAIN
FLM: FLOWERING LOCUS M
FT: FLOWERING LOCUS T
GA: gibberellic acid
GI: GIGANTEA
GUS: β-glucuronidase
HOS1: HIGH EXPRESSION OF OSMOTICALLY RESPONSIVE GENES 1
LHY: LATE ELONGATED HYPOCOTYL
L/D: light/dark
LD: long day
MBP: maltose binding protein
RGA1: REPRESSOR OF GA1
SD: short day
SOC1: SUPPRESSOR OF CONSTANS OVEREXPRESSION 1
SPY: SPINDLY
SVP: SHORT VEGETATIVE PAHSE
TOC1: TIMING OF CAB EXPRSSION 1
YFP: yellow fluorescence protein
