# Supplementary material for: LATE ELONGATED HYPOCOTYL regulates photoperiodic flowering via the circadian clock in Arabidopsis
Source: BMC Plant Biol. 2016 May 20;16:114. doi: 10.1186/s12870-016-0810-8 (PMC4875590; doi:10.1186/s12870-016-0810-8)
Supplement: Additional file 1: — Circadian rhythms in LHY-defective mutants. Ten-day-old plants grown on ½ X Murashige and Skoog-agar plates (hereafter, referred to as MS-agar plates) under long days (LDs, 16-h light and 8-h dark) were transferred to continuous light conditions at dawn (upper diagram). DAC, days after cold imbibition. Whole plants materials were harvested at the indicated zeitgeber time (ZT) points for total RNA extraction (lower panel). Rhythmic expression of CHLOROPHYLL A/B-BINDING PROTEIN 2 (CAB2) and CAROTENOID AND CHLOROPLAST REGULATION 2 (CCR2) genes, which exhibit circadian rhythmic expression patterns [41, 42], was examined by quantitative real-time RT-PCR (qRT-PCR). Biological triplicates were averaged. Bars indicate standard error of the mean. Two LHY-defective mutants (lhy-7 and lhy-20) were examined. (PDF 157 kb) [file 12870_2016_810_MOESM1_ESM.pdf]

Additional file 1

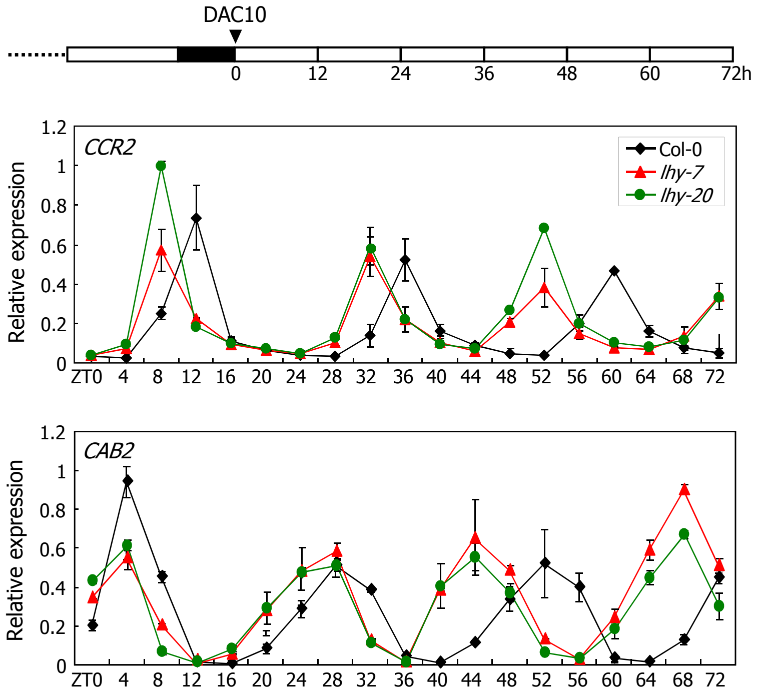

Additional file 1. Circadian rhythms in *LHY*-defective mutants.
Ten-day-old plants grown on ½ X Murashige and Skoog-agar plates (hereafter, referred to as MS-agar plates) under long days (LDs, 16-h light and 8-h dark) were transferred to continuous light conditions at dawn (upper diagram). DAC, days after cold imbibition. h, hour. Whole plants materials were harvested at the indicated zeitgeber time (ZT) points for total RNA extraction (lower panel). Rhythmic expression of *CHLOROPHYLL A/B-BINDING PROTEIN 2* (*CAB2*) and *CAROTENOID AND CHLOROPLAST REGULATION 2* (*CCR2*) genes, which exhibit circadian rhythmic expression patterns [41,42], was examined by quantitative real-time RT-PCR (qRT-PCR). Biological triplicates were averaged. Bars indicate standard error of the mean. Two *LHY*-defective mutants (*lhy-7* and *lhy-20*) were examined.
