# Supplementary material for: LATE ELONGATED HYPOCOTYL regulates photoperiodic flowering via the circadian clock in Arabidopsis
Source: BMC Plant Biol. 2016 May 20;16:114. doi: 10.1186/s12870-016-0810-8 (PMC4875590; doi:10.1186/s12870-016-0810-8)
Supplement: Additional file 2: — Expression of flowering time genes in lhy-7 mutant. Plants were grown under either LDs or short days (SDs, 8-h light and 16-h dark) for 10 days on MS-agar plates. Whole plants were harvested at ZT 8 for total RNA extraction. Transcript levels were examined by qRT-PCR. Biological triplicates were averaged and statistically treated using Student t-test (*P < 0.01). Bars indicate standard error of the mean. (PDF 131 kb) [file 12870_2016_810_MOESM2_ESM.pdf]

Additional file 2

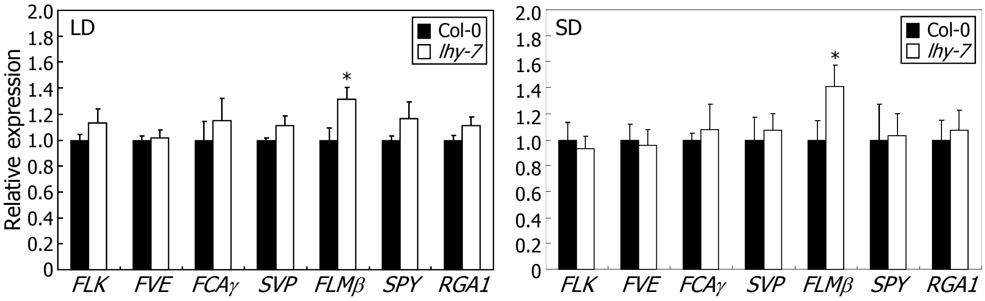

Additional file 2. Expression of flowering time genes in *lhy-7* mutant.
Plants were grown under either LDs or short days (SDs, 8-h light and 16-h dark) for ten days on MS-agar plates. Whole plants were harvested at ZT 8 for total RNA extraction. Transcript levels were examined by qRT-PCR. Biological triplicates were averaged and statistically treated using Student *t*-test (*$P < 0.01$). Bars indicate standard error of the mean.
