# Supplementary material for: LATE ELONGATED HYPOCOTYL regulates photoperiodic flowering via the circadian clock in Arabidopsis
Source: BMC Plant Biol. 2016 May 20;16:114. doi: 10.1186/s12870-016-0810-8 (PMC4875590; doi:10.1186/s12870-016-0810-8)
Supplement: Additional file 3: — Levels of LHY and CCA1 transcripts in 35S:LHY-MYC and 35S:MYC-CCA1 transgenic plants, respectively. Ten-day-old whole plants grown on MS-agar plates under LDs were harvested for total RNA extraction at the indicated ZT points. Transcript levels were examined by qRT-PCR. Biological triplicates were averaged and statistically treated (t-test, *P < 0.01). Bars indicate standard error of the mean. (PDF 121 kb) [file 12870_2016_810_MOESM3_ESM.pdf]

Additional file 3

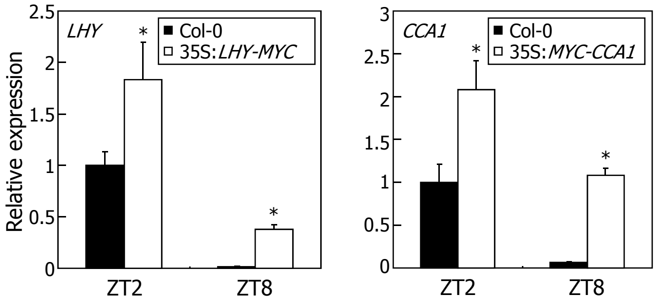

Additional file 3. Levels of *LHY* and *CCA1* transcripts in 35S:*LHY-MYC* and 35S:*MYC-CCA1* transgenic plants, respectively.

Ten-day-old whole plants grown on MS-agar plates under LDs were harvested at the indicated ZT points for total RNA extraction. Transcript levels were examined by qRT-PCR. Biological triplicates were averaged and statistically treated (*t*-test, $*P < 0.01$). Bars indicate standard error of the mean.
