# Supplementary material for: LATE ELONGATED HYPOCOTYL regulates photoperiodic flowering via the circadian clock in Arabidopsis
Source: BMC Plant Biol. 2016 May 20;16:114. doi: 10.1186/s12870-016-0810-8 (PMC4875590; doi:10.1186/s12870-016-0810-8)
Supplement: Additional file 4: — Functionality of 35S:MYC-CCA1 and 35S:LHY-MYC transgenic plants. A and B. Elongated hypocotyls. Plants were grown on MS-agar plates for 5 days under either LDs (A) or SDs (B). Measurements of 20 seedlings were averaged and statistically treated (t-test, *P < 0.01). Bars indicate standard error of the mean. C. Disruption of circadian rhythms. Expression patterns of CCR2 gene were examined as described in Additional file 1. Bars indicate standard error of the mean. D. Suppression of FT transcription. Plants were grown under LDs for 10 days on MS-agar plates. Whole plants were harvested at ZT16 for total RNA extraction. Transcript levels were examined as described in Additional file 2. Bars indicate standard error of the mean. (PDF 195 kb) [file 12870_2016_810_MOESM4_ESM.pdf]

Additional file 4

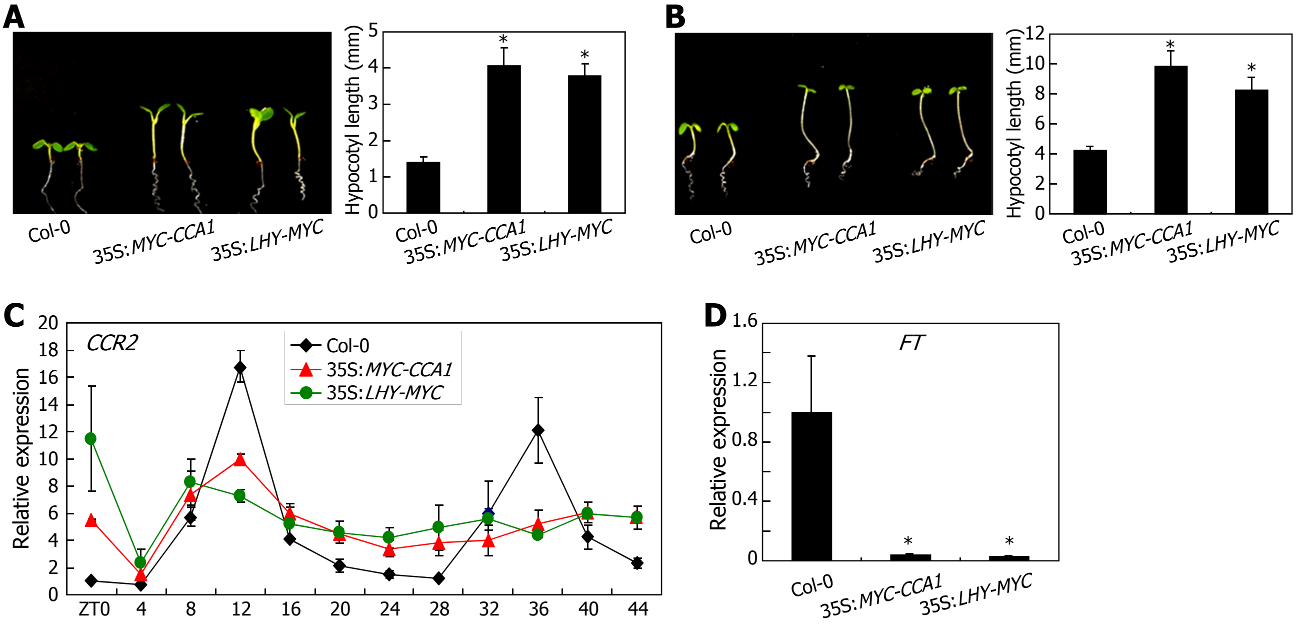

Additional file 4. Functionality of 35S:*MYC-CCA1* and 35S:*LHY-MYC* transgenic plants.
A and **B**. Elongated hypocotyls. Plants were grown on MS-agar plates for 5 days under either LDs (**A**) or SDs (**B**). Measurements of 20 seedlings were averaged and statistically treated (*t*-test, $^*P < 0.01$). Bars indicate standard error of the mean.
C. Disruption of circadian rhythms. Expression patterns of *CCR2* gene were examined as described in **Additional file 1**. Bars indicate standard error of the mean.
D. Suppression of *FT* transcription. Plants were grown under LDs for 10 days on MS-agar plates. Whole plants were harvested at ZT16 for total RNA extraction. Transcript levels were examined as described in **Additional file 2**. Bars indicate standard error of the mean.
