# Supplementary material for: LATE ELONGATED HYPOCOTYL regulates photoperiodic flowering via the circadian clock in Arabidopsis
Source: BMC Plant Biol. 2016 May 20;16:114. doi: 10.1186/s12870-016-0810-8 (PMC4875590; doi:10.1186/s12870-016-0810-8)
Supplement: Additional file 5: — Recombinant proteins used for electrophoretic mobility shift assay (EMSA). Recombinant maltose-binding protein (MBP) and MBP-CCA1 fusion protein were prepared in E. coli cells and affinity-purified. Protein quality was verified by running on 10 % SDS-PAGE and Coomassie brilliant blue staining. The arrow and arrowhead indicate full-size MBP and MBP-CCA1 proteins, respectively. SM, size marker. kDa, kilodalton. (PDF 263 kb) [file 12870_2016_810_MOESM5_ESM.pdf]

Additional file 5

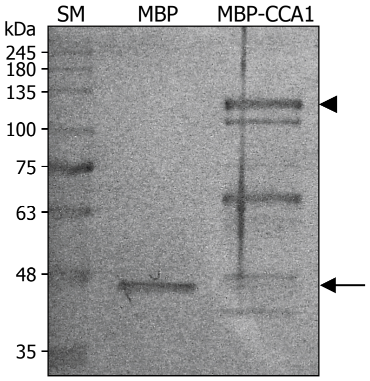

Additional file 5. Recombinant proteins used for electrophoretic mobility shift assay (EMSA). Recombinant maltose-binding protein (MBP) and MBP-CCA1 fusion protein were prepared in *E. coli* cells and affinity-purified. Protein quality was verified by running on 10% SDS-PAGE and Coomassie brilliant blue staining. The arrow and arrowhead indicate full-size MBP and MBP-CCA1 proteins, respectively. SM, size marker. kDa, kilodalton.
