# Supplementary material for: LATE ELONGATED HYPOCOTYL regulates photoperiodic flowering via the circadian clock in Arabidopsis
Source: BMC Plant Biol. 2016 May 20;16:114. doi: 10.1186/s12870-016-0810-8 (PMC4875590; doi:10.1186/s12870-016-0810-8)
Supplement: Additional file 6: — EMSA on binding of MBP and MBP-CCA1 proteins to conserved sequences in FT locus. Recombinant MBP and MBP-CCA1 fusion proteins were prepared as described in Additional file 5. Radio-labelled CCA1-binding sequence (CBS) and evening element (EE) DNA fragments, which were described in Fig. 4a, were used. A. EMSA on MBP binding to DNA fragments. (+) and (−) indicate assays with or without MBP protein. Note that MBP alone does not bind to the CBS and EE sequences. B. EMSA on MBP-CCA1 binding to DNA fragments. The core sequences of CBS and EE were mutated, resulting in mCBS and mEE, respectively. Excess amounts (50X, 100X) of unlabeled DNA fragments were added as competitors. (PDF 182 kb) [file 12870_2016_810_MOESM6_ESM.pdf]

Additional file 6

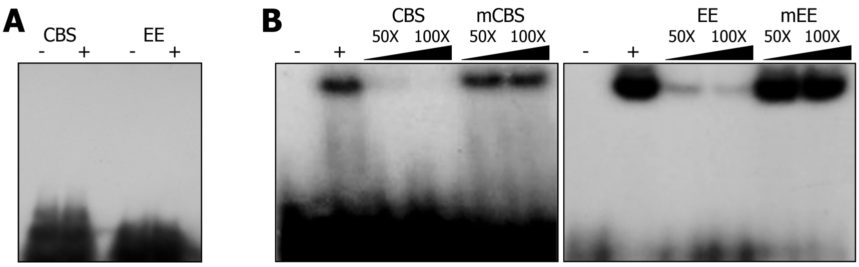

Additional file 6. EMSA on binding of MBP and MBP-CCA1 to conserved sequences in *FT* locus. Recombinant MBP and MBP-CCA1 fusion proteins were prepared as described in **Additional file 5**. Radio-labelled CCA1-binding sequence (CBS) and evening element (EE) DNA fragments, which were described in **Figure 4A**, were used.

A. EMSA on MBP binding to DNA fragments. (+) and (-) indicate assays with or without MBP protein. Note that MBP alone does not bind to the CBS and EE sequences.

B. EMSA on MBP-CCA1 binding to DNA fragments. The core sequences of CBS and EE were mutated, resulting in mCBS and mEE, respectively. Excess amounts (50X, 100X) of unlabeled DNA fragments were added as competitors.
