# Supplementary material for: LATE ELONGATED HYPOCOTYL regulates photoperiodic flowering via the circadian clock in Arabidopsis
Source: BMC Plant Biol. 2016 May 20;16:114. doi: 10.1186/s12870-016-0810-8 (PMC4875590; doi:10.1186/s12870-016-0810-8)
Supplement: Additional file 7: — ChIP assays on LHY binding to GI promoter. Chromatins were prepared from 7-day-old whole plants grown on MS-agar plates and immunoprecipitated using an anti-MYC antibody. Fragmented genomic DNA was eluted from the protein-DNA complexes and subjected to quantitative PCR. Biological triplicates were averaged and statistically treated using Student t-test (*P < 0.01). Bars indicate standard error of the mean. GI (NB) amplifies a downstream sequence region of GI gene, and GI (CBS) amplifies a sequence region containing CBS in the GI promoter, which has been described previously [38]. (PDF 122 kb) [file 12870_2016_810_MOESM7_ESM.pdf]

Additional file 7

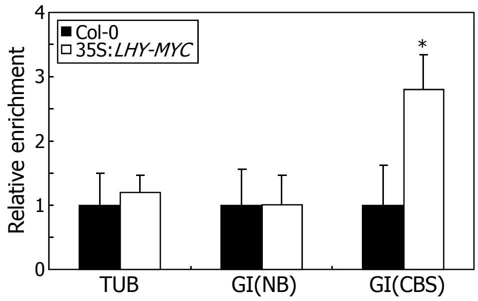

Additional file 7. ChIP assays on LHY binding to *GI* promoter.
Chromatins were prepared from seven-day-old whole plants grown on MS-agar plates and immunoprecipitated using an anti-MYC antibody. Fragmented genomic DNA was eluted from the protein-DNA complexes and subjected to quantitative PCR. Biological triplicates were averaged and statistically treated using Student *t*-test (*$P < 0.01$). Bars indicate standard error of the mean. GI(NB) amplifies a downstream sequence region of *GI* gene, and GI(CBS) amplifies a sequence region containing CBS in the *GI* promoter, which has been described previously [38].
