# Supplementary material for: LATE ELONGATED HYPOCOTYL regulates photoperiodic flowering via the circadian clock in Arabidopsis
Source: BMC Plant Biol. 2016 May 20;16:114. doi: 10.1186/s12870-016-0810-8 (PMC4875590; doi:10.1186/s12870-016-0810-8)
Supplement: Additional file 8: — Expression of flowering genes in lhy-7 mutant under short days of 20-h total duration. Plants were grown for 10 days under short-day cycles of either 24-h (8-h light and 16-h dark) or 20-h (6.7-h light and 13.3-h dark) total duration. Whole plant materials were harvested throughout the % light-dark (L/D) cycles. Transcript levels were examined by qRT-PCR in Col-0 plants (A) and lhy-7 mutant (B). Biological triplicates were averaged. Bars indicate standard error of the mean. h, hour. (PDF 133 kb) [file 12870_2016_810_MOESM8_ESM.pdf]

Additional file 8

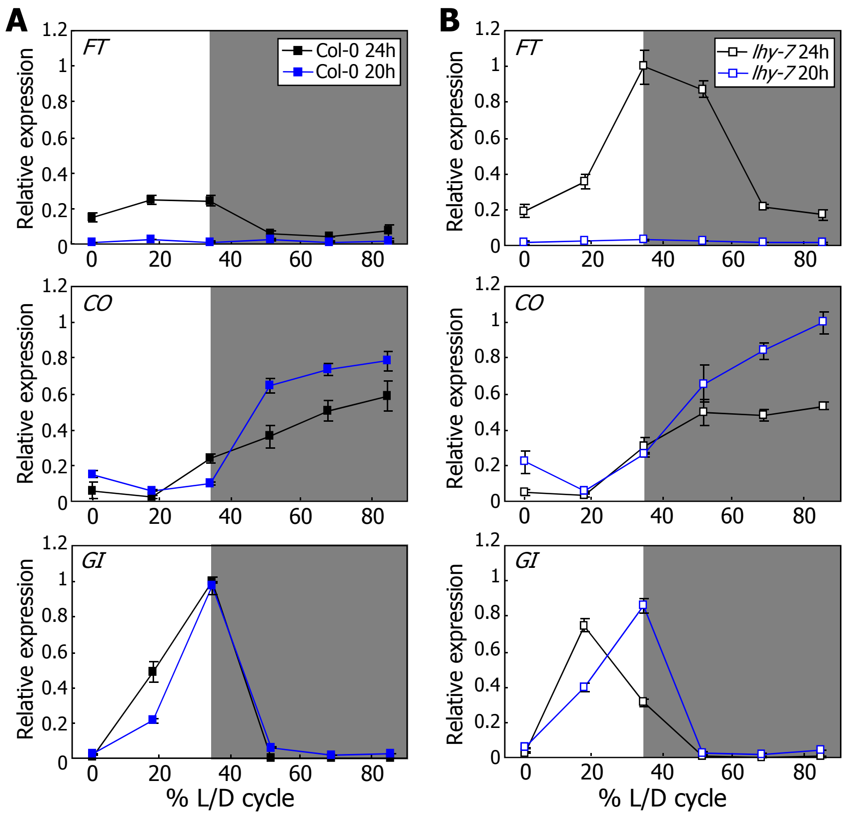

Additional file 8. Expression of flowering genes in *lhy-7* mutant under short days of 20-h total duration.

Plants were grown for ten days under short-day cycles of either 24-h (8-h light and 16-h dark) or 20-h (6.7-h light and 13.3-h dark) total duration. Whole plant materials were harvested throughout the % light-dark (L/D) cycles. Transcript levels were examined by qRT-PCR in Col-0 plants (**A**) and *lhy-7* mutant (**B**). Biological triplicates were averaged. Bars indicate standard error of the mean. h, hour.
