# Supplementary material for: LATE ELONGATED HYPOCOTYL regulates photoperiodic flowering via the circadian clock in Arabidopsis
Source: BMC Plant Biol. 2016 May 20;16:114. doi: 10.1186/s12870-016-0810-8 (PMC4875590; doi:10.1186/s12870-016-0810-8)
Supplement: Additional file 9: — Expression of flowering genes in lhy-7 mutant under SDs of 20-h total duration. Plants were grown for 10 days under short-day cycles of 20-h total duration (6.7-h light and 13.3-h dark). Whole plant materials were harvested for total RNA extraction. Transcript levels were examined by qRT-PCR. Biological triplicates were averaged and statistically treated using Student t-test (*P < 0.01). Bars indicate standard error of the mean. (PDF 130 kb) [file 12870_2016_810_MOESM9_ESM.pdf]

Additional file 9

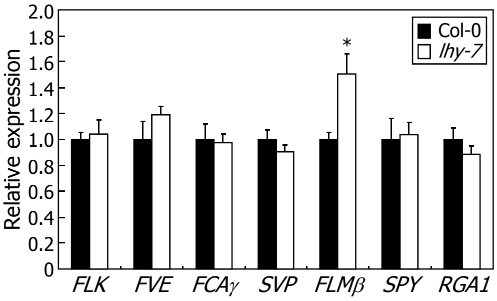

Additional file 9. Expression of flowering genes in *lhy-7* mutant under SDs of 20-h total duration. Plants were grown for ten days under short-day cycles of 20-h total duration (6.7-h light and 13.3-h dark). Whole plant materials were harvested for total RNA extraction. Transcript levels were examined by qRT-PCR. Biological triplicates were averaged and statistically treated using Student *t*-test (*$P < 0.01$). Bars indicate standard error of the mean.
