# Supplementary material for: LATE ELONGATED HYPOCOTYL regulates photoperiodic flowering via the circadian clock in Arabidopsis
Source: BMC Plant Biol. 2016 May 20;16:114. doi: 10.1186/s12870-016-0810-8 (PMC4875590; doi:10.1186/s12870-016-0810-8)
Supplement: Additional file 10: — Primers used in qRT-PCR, RT-PCR, and ChIP-qPCR. F, forward primer; R, reverse primer. (PDF 90 kb) [file 12870_2016_810_MOESM10_ESM.pdf]

Additional file 10

Primer	Usage	Sequence
eIF4a-F	qRT-PCR	5′-TGACCACACAGTCTCTGCAA
eIF4a-R	qRT-PCR	5′-ACCAGGGAGACTTGTTGGAC
TUB-F	qRT-PCR, RT-PCR	5′-TTTGTGCTCATCTTGCCACGGAAC
TUB-R	qRT-PCR, RT-PCR	5′-CTCAAGAGGTTCTCAGCAGTACC
LHY-F	qRT-PCR	5′-ATGGATACTAATACATCTGGAGAAGA
LHY-R	qRT-PCR	5′-TCATGTAGAAGCTTCTCCTTCCA
CCA1-F	qRT-PCR	5′-GATCTGGTTATTAAGACTCGGAAGCCATATAC
CCA1-R	qRT-PCR	5′-GCCTCTTTCTCTACCTTGGAGA
FT-F	qRT-PCR	5'-GGTGGAGAAGACCTCAGGAA
FT-R	qRT-PCR	5'-ATTGTAGAAAACTGCGGCCA
SOC1-F	qRT-PCR	5′-GGATCTCATGAAAGCGAAGTTT
SOC1-R	qRT-PCR	5′-TCACTTTCTTGAAGAACAAGGTA
CO-F	qRT-PCR	5'-ACGCCATCAGCGAGTTCC
CO-R	qRT-PCR	5'-AAATGTATGCGTTATGGTTAATGG
GI-F	qRT-PCR	5′-TGGCGATGTTTGTCCTTCAT
GI-R	qRT-PCR	5′-AGCCCATTGCTCCGAATAGT
FKF1-F	qRT-PCR	5'-GTCGTAACTGTCGATTCCTACA
FKF1-R	qRT-PCR	5'-ATCTCCAGTGTTCCAGTTATCT
FLC-F	qRT-PCR	5′-CCCTCTCCGTGACTAGAGCC
FLC-R	qRT-PCR	5′-TGGGAGAGTCACCGGAAGAT
CAB2-F	qRT-PCR	5′-GTTTGTGTTTGTGGTGGATGGT
CAB2-R	qRT-PCR	5′-CGGAGTGAACCCAAGAACTGA
CCR2-F	qRT-PCR	5′-GCTCTTGAGACTGCCTTCGCTC
CCR2-R	qRT-PCR	5′-CTCGTTAACAGTGATGCTACGG
FLK-F	qRT-PCR	5′-GCCTTCAAAGGTGTCAACAAGA
FLK-R	qRT-PCR	5′-CAGGTAAATCCTCTGATCCAAGAA
FVE-F	qRT-PCR	5′-TCCTGACAGGGTCAGCAGAC
FVE-R	qRT-PCR	5′-TCAGGAGACCACTGAACACAAA
FCAγ-F	qRT-PCR	5′-GCTCTTGTCGCAGCAAACTC
FCAγ-R	qRT-PCR	5′-GATCCAGCCCACTGTTGTTTAC
SVP-F	qRT-PCR	5′-CAAGGAACGCAACTAACGGA
SVP-R	qRT-PCR	5′-AGTGTCGGAGCTCTCGGAGT
FLMβ-F	qRT-PCR	5′-CATGCTGATGAACTTAGAGCCTTAGATC
FLMβ-R	qRT-PCR	5′-CAGCAACGTATTCTTTCCCAT
SPY-F	qRT-PCR	5′-CCTGTTTGTCCAACACCTGC
SPY-R	qRT-PCR	5′-CTGCCTAATGCTATCGCAGC
RGA1-F	qRT-PCR	5′-TACATCGACTTCGACGGGTA
RGA1-R	qRT-PCR	5′-GTTGTCGTCACCGTCGTTC
FT(CBS)-F	ChIP-qRCR	5′-TCGTGCAAATGGATGGTTAGT
FT(CBS)-R	ChIP-qPCR	5′-TCCGAACTCAAATACGCAAAA
FT(EE)-F	ChIP-qPCR	5′-GGGATTTTTCTTTGTTCCTCCT
FT(EE)-R	ChIP-qPCR	5′-AAGGCTGGCTTGAATATCAGAA
FT(NB)-F	ChIP-qPCR	5′-GCGCCAGAACTTCAACACTC
FT(NB)-R	ChIP-qPCR	5′-AAGTCTTCTTCCTCCGCAGC
TOC1(EE)-F	ChIP-qPCR	5′-CTTCTTATCTTGTATCTTACCAC
TOC1(EE)-R	ChIP-qPCR	5′-GAATTGGACGGTGGAGATTAAGTC
GI(NB)-F	ChIP-qPCR	5′-CTGGAAAGCCGATATCCAAA
GI(NB)-R	ChIP-qPCR	5′-AAGTGGGTGCTCGTTATTGG
GI(CBS)-F	ChIP-qPCR	5′-ACCTTTTTCGTGGTTTTTGG
GI(CBS)-R	ChIP-qPCR	5′-TTCAGAAGAACAAAATCTCAATCA

Additional file 10. Primers used in qRT-PCR, RT-PCR, and ChIP-qPCR.
F, forward primer; R, reverse primer.
